# Bioactivity-guided identification and cell signaling technology to delineate the immunomodulatory effects of Panax ginseng on human promonocytic U937 cells

**DOI:** 10.1186/1479-5876-7-34

**Published:** 2009-05-14

**Authors:** Davy CW Lee, Cindy LH Yang, Stanley CC Chik, James CB Li, Jian-hui Rong, Godfrey CF Chan, Allan SY Lau

**Affiliations:** 1 Cytokine Biology Group, Department of Paediatrics and Adolescent Medicine, The University of Hong Kong, Pokfulam, Hong Kong Special Administrative Region, PR China; 2 Molecular Chinese Medicine Laboratory, Li Ka Shing Faculty of Medicine, The University of Hong Kong, Pokfulam, Hong Kong Special Administrative Region, PR China

## Abstract

**Background:**

Ginseng is believed to have beneficial effects against human diseases, and its active components, ginsenosides, may play critical roles in its diverse physiological actions. However, the mechanisms underlying ginseng's effects remain to be investigated. We hypothesize some biological effects of ginseng are due to its anti-inflammatory effects.

**Methods:**

Human promonocytic U937 cells were used to investigate the immunomodulatory effects of ginseng following TNF-α treatment. A global gene expression profile was obtained by using genechip analysis, and specific cytokine expression was measured by quantitative RT-PCR and ELISA. HPLC was used to define the composition of ginsenosides in 70% ethanol-water extracts of ginseng. Activation of signalling kinases was examined by Western blot analysis.

**Results:**

Seventy percent ethanol-water extracts of ginseng significantly inhibited the transcription and secretion of CXCL-10 following TNF-α stimulation. Nine ginsenosides including Rb_1_, Rb_2_, Rc, Rd, Re, Rf, Rg_1_, Rg_3 _and Rh_1 _were identified in our extract by HPLC. Seven out of nine ginsenosides could significantly inhibit TNF-α-induced CXCL-10 expression in U937 cells and give comparable inhibition of CXCL-10 transcription to those with the extract. However, the CXCL-10 suppressive effect of individual ginsenosides was less than that of the crude extract or the mixture of ginsenosides. The CXCL-10 suppression can be correlated with the inactivation of ERK1/2 pathways by ginseng.

**Conclusion:**

We showed ginseng suppressed part of the TNF-α-inducible cytokines and signalling proteins in promonocytic cells, suggesting that it exerts its anti-inflammatory property targeting at different levels of TNF-α activity. The anti-inflammatory role of ginseng may be due to the combined effects of ginsenosides, contributing in part to the diverse actions of ginseng in humans.

## Background

*Panax ginseng *(ginseng) has been used as a herbal remedy in ancient China and Asian countries for thousands of years and became popular in Western countries during the last two decades [1]. Ginseng roots contain multiple active constituents including ginsenosides, polysaccharides, peptides, polyacetylenic alcohols and fatty acids that have been shown to have different effects on carbohydrate and lipid metabolism as well as on the function of neuroendocrine, immune, cardiovascular and central nervous systems in humans [1,2]. Previous studies have shown that ginseng and its active components are potent immunomodulators. Their immunomodulatory effects are mostly due to its regulation of cytokine production and phagocytic activities of monocytes/macrophages and dendritic cells, as well as activation of T- and B- lymphocytes [3-8].

In addition, ginseng has been shown to have potent regulatory effects on the inflammatory cascade. Ginsan, a polysaccharide extract from ginseng, enhances the phagocytic activity of macrophages in mice infected with *Staphylococcus aureus *[9]. Ginsan also inhibits the production of proinflammatory cytokines including tumour necrosis factor-α (TNF-α), interleukin-1β (IL-1β), IL-6, IL-12, IL-18 and interferon-γ (IFN-γ) by suppressing the activity of mitogen activated protein kinases (MAPK) including p38 MAPK and JNK, and the transcription factor nuclear factor-kappaB (NF-κB). The ginseng root extract stimulates the inducible nitric oxide synthase (iNOS) activity in RAW264.7 murine macrophages [10].

Ginsenosides, the steroid saponins, are major biologically active compounds of ginseng. Over 30 ginsenosides have been identified to date [11]. Studies indicate that ginsenosides and their metabolites are responsible for many of the diverse physiological actions including the anti-inflammatory effects of ginseng. For example, ginsenoside Rh_1 _reduces histamine release from rat peritoneal mast cells and the IgE-mediated passive cutaneous anaphylaxis reaction in mice [12]. Rh_1 _and 20(S)-Protopanaxatriol inhibit the LPS-induced expression of iNOS and cyclooxygenase-2 (COX-2) in RAW264.7 cells through the inactivation of NF-κB [12,13]. Ginsenoside Rg_3 _inhibits the expression of 12-O-tetradecanoylphorbol-13-acetate-induced COX-2 as well as activation of NF-κB and AP-1 in mouse skin and human pro-myelocytic leukemia cells [14].

Proinflammatory cytokine TNF-α has been shown to play a central role in the pathogenesis of both acute infectious diseases and chronic inflammatory conditions [15,16]. Production of TNF-α by the host is one of the important defence mechanisms against bacterial, viral or parasitic infections. However, excess local TNF-α production can promote the neighbouring tissue damage and inflammation through the induction of chemokines and other factors [15]. Hence, different anti-TNF-α therapies have been developed for patients with chronic inflammatory diseases including rheumatoid arthritis, Crohn's disease and psoriasis [15,17].

To investigate the immunomodulatory effects of *Panax ginseng*, genechip analysis was used to examine the gene expression profile of TNF-α-treated human monocytic U937 cells with or without pre-treatment with a *Panax ginseng *extract (PGSE). The semi-quantitative results on specific cytokines were validated by quantitative RT-PCR and ELISA. Moreover, the composition of ginsenosides in the PGSE was determined by using high performance liquid chromatography (HPLC) analysis. The effects of individual ginsenoside or mixtures of HPLC-defined ginsenosides on U937 cells with subsequent TNF-α treatment were examined by quantitative RT-PCR analysis. Our results may contribute to the understanding of the molecular mechanisms of the immunomodulatory effect of ginseng and ginsenosides on TNF-α-mediated inflammatory diseases.

## Methods

### Preparation of 70% ethanol-water extracts of ginseng (PGSE)

The *Panax ginseng *extract was provided by Prof Wang Jianxin (Shanghai Institute of Chinese Materia Medica, PRChina). Briefly, the crude plant material of ginseng was cut into slices of 1 to 3 mm, and then placed in a flask that was heated with 70% ethanol-water under reflux for 6 hours. The experiment was repeated twice. The ratio of the plant material to the menstruum was 1:10. The resultant extract was concentrated by evaporation and then dried by lyophilization to obtain PGSE at a yield between 20 to 25% (w/w, dried extract/crude herb). The extract was grinded and then passed through an 80 mesh screen.

### High performance liquid chromatography analysis of PGSE

Ginsenosides standards were purchased from Chromadex. HPLC analysis on the composition of ginsenosides in PGSE (2 mg in 1.5 ml of milli-Q water) was performed by using an Agilent 1200 liquid chromatography system that was equipped with a quaternary solvent delivery system, an autosampler and photodiode array detector. A reversed-phase column, Lichrospher C_18 _(250 mm × 4.6 mm i.d., 5 μm), was used for all separations. The gradient program, modified from a previous report [18], consisted of (A) water and (B) acetonitrile at a flow of 1 mL/min, as follows: 0–6 min, 21–22% B; 6–7 min, 22–23% B; 7–25 min, 23–24% B; 25–30 min, 24–30% B; 30–40 min, 30–32% B; 40–45 min, 32–50% B; 45–60 min, 50–65% B; 60–61 min, 65–100% B; and 61–65 min, back to 21% B before the next injection. The injection volume was 15 μl and the UV detection wavelength was performed at 203 nm for all ginsenosides and PGSE.

### Cell culture

The human promonocytic U937 cells [19] were obtained from American Type Culture Collection (ATCC accession no. CRL-1593.2™) and were cultured in RPMI 1640 medium (Invitrogen) supplemented with 10% foetal bovine serum (Invitrogen), penicillin (100 U/ml) and streptomycin (100 μg/ml) in a 5% CO_2 _incubator at 37°C. Cells were incubated with TNF-α (20 units/ml) for 2 hours with or without the pre-treatment of PGSE for 24 hours and harvested for genechip analysis. The PGSE concentrations used in our report are based on previous studies of ginseng by other investigators [20,21] and verified by our cytotoxicity tests. The effective doses of ginsenosides in other groups' *in vitro *studies ranged from 10 – 100 μM or 0.01 – 0.1 mg/ml. Similarly, the concentrations of individual ginsenosides in 3 mg PGSE used in our experiments ranged from 0.01 to 0.14 mg/ml (Table 1). Therefore, at these low concentrations, it is conceivable that the ginsenoside content of 3 mg/ml PGSE is achievable *in vivo*. In addition, we determined the cytotoxic effects of PGSE at 3 mg/ml by trypan blue exclusion assay. The viability of cells was over 90% after incubating U937 cells with the PGSE for 48 hours.

**Table 1:** Distribution of ginsenosides in *Panax ginseng *extract.

GS	Amount of GS in 3 mg of PGSE (mg)	Molarity (mM)	Percentage of GS in 3 mg of PGSE (w/w)
Rb_1_	0.14	0.13	4.7%
Rb_2_	0.07	0.06	2.5%
Rc	0.08	0.07	2.8%
Rd	0.04	0.04	1.3%
Re	0.07	0.07	2.2%
Rf	0.01	0.01	0.4%
Rg_1_	0.12	0.15	4.0%
Rg_3_	0.02	0.03	0.6%
Rh_1_	0.01	0.02	0.3%
Rh_2_	0.00	0.00	0.0%
			Total: 18.8%

The amount of ginsenosides was determined using HPLC (n = 2); GS, ginsenosides; *Panax ginseng *extract, PGSE.

### Cytotoxicity test of PGSE

Cytotoxic effects of PGSE on U937 cells were examined by incubating 3 mg/ml of PGSE for 48 hours and the cell viability was determined by using trypan blue exclusion test. There is no significant sign of cytotoxicity found at 3 mg/ml of PGSE.

### Limulus amebocyte lysate test

The amount of bacterial endotoxin in PGSE was measured by Pyrotell Limulus amebocyte lysate assay kit (Associates of Cape Cod) according to the manufacturer's protocol. Briefly, 0.2 ml of various concentrations of PGSE was added to a single test vial of Pyrotell. The reaction mixture was incubated at 37°C for 60 min and then inverted to observe the gel formation. Positive result is indicated by the formation of an intact gel which does not collapse upon inversion. The levels of endotoxin in PGSE at 10 mg/ml were lower than the detection limit of the test (<0.05 ng/ml) indicating that the biological effects of PGSE are not due to endotoxin contamination.

### Isolation of RNA and microarray analysis

U937 cells (1 × 10^6^) were pretreated with or without 3 mg/ml PGSE for 24 hours followed by 20 units/ml TNF-α for 2 hours and Genechip analysis was followed by using Affymetrix's protocol. Briefly, total cellular RNA was extracted using TRIzol (Invitrogen) and further purified by RNeasy cleanup kit (Qiagen) according to the manufacturer's instructions. The RNA integrity was determined by the ratio of 28S/18S ribosomal RNA using Agilent 2100 Bioanalzyer. For genechip analysis, total RNA (1 μg) were reverse transcribed to the first-stranded cDNA by using oligo (dT) linked-T7 RNA polymerase promoter sequence and the double-stranded cDNA was synthesized by using RT Kit (Invitrogen). The biotin labelled-cRNA was generated by *in vitro *transcription kit (Invitrogen), purified by RNeasy mini columns (Qiagen), denatured and 15 μg cRNA was hybridized to Human Genome U133 Plus 2.0 arrays (Affymetrix). Then, the arrays were stained with a streptavidin-phycoerythrin conjugate and visualized with GeneArray scanner (Agilent). The genechip data were analyzed by using Agilent Genespring GX and Affymetrix GeneChip Operating Softwares (GCOS). The signal intensity of each gene was firstly normalized with the total intensity of all genes from the genechip, and then the normalized signal of each treatment was compared with the mock-treatment to determine the relative fold changes of gene expression. The threshold level for up- or down-regulation of gene expression was the level of changes ≥2-fold.

### Quantitative RT-PCR analysis

U937 cells were treated as described in genechip analysis and the procedures of quantitative RT-PCR analysis were described in our previous studies [22-24]. Briefly, DNase-treated RNA samples were reverse transcribed using TaqMan reverse transcription reagent kit (Applied Biosystems) and the levels of CXCL-10, IL-8 and TNFAIP3 mRNA as well as the reference gene 18S rRNA were assayed by the gene-specific TaqMan gene expression assays (Applied Biosystems). All samples and controls were run in triplicates on an ABI 7500 Real-time PCR system. The quantitative RT-PCR data was analyzed by the comparative cycle number threshold method and the fold inductions of samples were compared with the untreated samples.

### ELISA

U937 cells were pre-treated with or without PGSE (3 mg/ml) for 24 hours prior to TNF-α (20 units/ml) stimulation for 16 hours. After treatment, the levels of CXCL-10 and IL-8 in culture supernatant were measured by using the respective commercially available specific ELISA kits (R&D Systems).

### Preparation of protein lysate

U937 cells were pre-treated with or without PGSE (1 or 3 mg/ml) for 24 hours followed by TNF-α (20 units) stimulation for 2 hours. To prepare the whole cell lysate, cells were washed with PBS and lysed with ice-cold lysis buffer containing 1% Triton X-100, 25 mM HEPES, 5 mM EDTA, 100 mM NaCl, 0.1 mg/ml PMSF, 2 μg/ml aprotinin, 1 mM sodium orthovanadate, 2 μg/ml pepstatin, 2 μg/ml leupeptin, 50 mM sodium fluoride and 10 mM beta-glycerophosphate for 20 min on ice. The total protein was harvested by centrifugation at 13000 rpm for 10 min at 4°C. The supernatants were stored as aliquots at -70°C.

### Western analysis

Protein concentration was determined by BCA protein assay reagent kit (Pierce) according to the supplier's procedures. Thirty micrograms of total protein lysate were separated by 10% SDS-PAGE, electroblotted onto nitrocellulose membranes (Schleicher & Schuell), and then probed with anti-phospho-ERK1/2 polyclonal antibodies or anti-phospho-p38 MAPK polyclonal antibodies (Cell signaling). Control blots were immunoblotted with anti-ERK1/2 or anti-p38 MAPK polyclonal antibodies for whole cell lysates. Immuoblots were then incubated with HRP-conjugated anti-rabbit antibodies (BD Bioscience). Finally, the blot was incubated with the Enhanced Chemiluminescence System (GE Healthcare) to detect the target proteins.

### Data analysis

All data are presented as the mean ± standard deviation (SD) obtained from at least three separate experiments and statistically analyzed by two-tailed, paired *t*-test. The statistical significance was defined as *p < 0.05; ^†^p < 0.01; ^ψ^p < 0.005.

## Results

### Immunomodulatory effects of PGSE on U937 cells stimulated by TNF-α

To investigate the immunomodulatory activity of ginseng, U937 cells were treated with PGSE and followed by TNF-α stimulation. The gene expression profiles of total cellular RNA were examined by Affymetrix genechip analysis and the data were analyzed by using the Affymetrix GCOS and Genespring GX softwares as described in Methods. To increase the stringency of the analysis, we combined the gene lists from the two software analyses. Only the genes found in both gene lists were reported in this study. Cells with TNF-α or PGSE treatment only were included, and the fold induction of cytokines in cells with treatment was normalized with that of the untreated cells.

**Figure 1:**
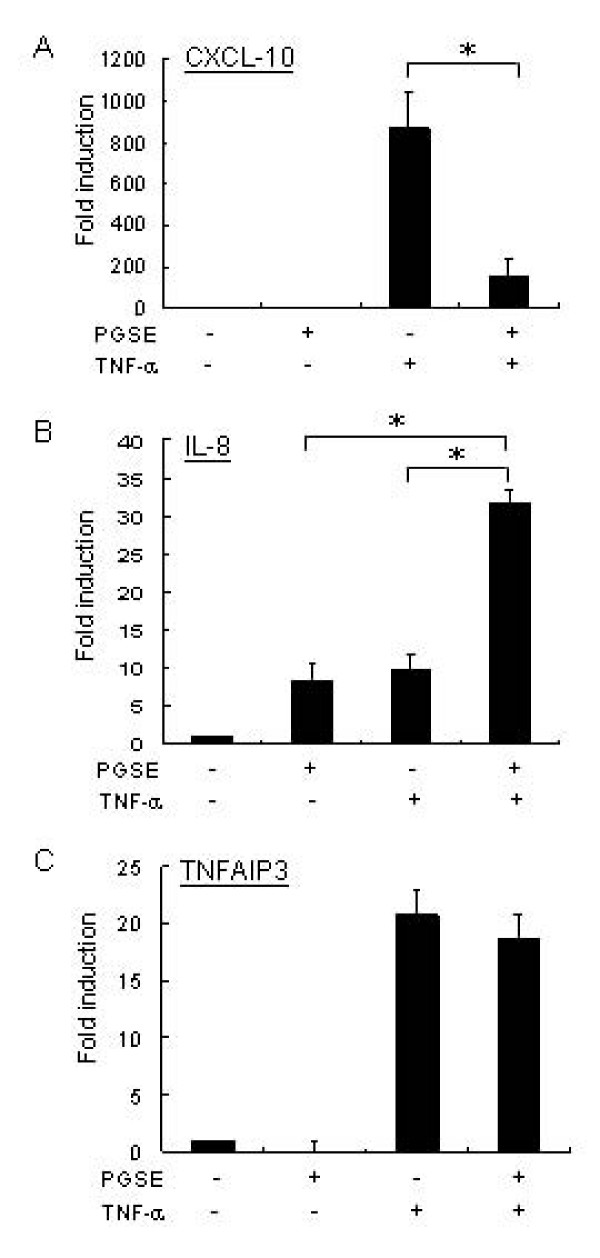
**Quantitative RT-PCR analysis of TNF-α regulated genes in U937 cells after sequential treatment with PGSE and TNF-α**. U937 cells (1 × 10^6^) were pretreated with or without 3 mg/ml PGSE for 24 hours and followed by 20 units/ml of TNF-α for 2 hours. DNase-treated RNA samples were reverse transcribed and the levels of mRNA induction of (A) CXCL-10, (B) IL-8 and (C) TNFAIP3 as well as the reference gene 18S rRNA were determined by gene-specific TaqMan assays as described in Methods. The levels of induction were relative to the untreated cells. Values represent the average ± SD of three independent experiments and statistically analyzed by two tailed, paired t-test. *: p < 0.05. PGSE, 70% ethanol-water extracts of ginseng; CXCL-10, interferon gamma-inducible protein-10; IL-8, interleukin-8; TNFAIP3, TNF-α-induced protein 3.

**Figure 2:**
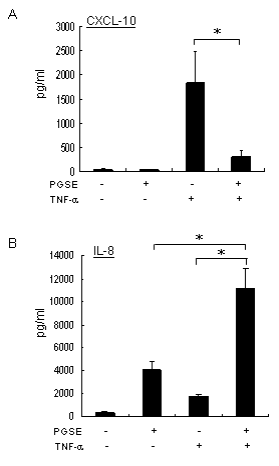
**Quantification of CXCL-10 and IL-8 in culture supernatant of U937 cells by ELISA**. U937 cells were pretreated with or without 3 mg/ml PGSE for 24 hours prior to 20 units/ml TNF-α stimulation for 16 hours. After treatment, the level of CXCL-10 in culture supernatants was measured by specific ELISA kit according to the supplier's procedures. Values represent the average ± SD of three independent experiments and statistically analyzed by two tailed, paired t-test. *: p < 0.05. PGSE, 70% ethanol-water extracts of ginseng; CXCL-10, interferon gamma-inducible protein-10; IL-8, interleukin-8.

Following the sequential treatment of PGSE and TNF-α, we found that 102 upregulated genes and 64 downregulated genes were repeatedly shown in the gene list of two analyses (data not shown). To determine the effects of PGSE on TNF-α signalling pathways, the TNF-α-inducible cytokines and signalling proteins were grouped and summarized in Table 2. Our results showed that PGSE suppressed the transcription of TNF-α inducible genes including CXCL-10, NF-κB inhibitor alpha (IκB-α), G protein-coupled receptor 84, phosphodiesterase 4B, CXCL-11 and CCL-3 in U937 cells. In contrast, PGSE enhanced the transcription of IL-8 with TNF-α, but it did not affect the transcription of CXCL-2, CCL-2, IL-18 receptor, IL-1β and TNF-α-induced protein 3 (TNFIP3). The genechip results of CXCL-10 and IL-8 were validated by quantitative RT-PCR and ELISA. Consistently, PGSE showed inhibition on TNF-α-induced CXCL-10 expression (Figures 1A and 2A) but augmentation of TNF-α-induced IL-8 expression (Figures 1B and 2B). By contrast, there was no significant change of the transcription of TNFIP3 in TNF-α-treated U937 cells with PGSE treatment (Figure 1C).

**Table 2:** Summary of the effect of *Panax ginseng *extract (PGSE) on TNF-α regulated genes

Mock	TNF	PGSE+TNF	PGSE	Gene symbol	Description
1.0	53.55	5.61	1.35	CXCL10	Chemokine (C-X-C motif) ligand 10
1.0	13.04	11.03	0.82	TNFAIP3	TNF-α-induced protein 3
1.0	12.40	12.15	1.93	CXCL2	Chemokine (C-X-C motif) ligand 2
1.0	12.28	8.64	1.14	NFKBIA	NK-κB inhibitor, alpha
1.0	11.17	9.75	0.88	TNFAIP3	TNF-α-induced protein 3
1.0	7.47	6.04	0.99	IER3	Immediate early response 3
1.0	7.21	2.35	0.86	GPR84	G protein-coupled receptor 84
1.0	7.18	4.90	1.20	NFKBIZ	NF-κB inhibitor, zeta
1.0	6.22	4.37	0.62	PDE4B	Phosphodiesterase 4B
1.0	6.06	13.38	5.39	IL8	Interleukin 8
1.0	6.05	2.70	0.83	TNFAIP6	TNF-α-induced protein 6
1.0	4.12	1.65	1.10	TNFAIP6	TNF-α-induced protein 6
1.0	3.73	11.23	4.38	IL8	Homo sapiens IL8 C-terminal variant
1.0	3.11	2.23	0.81	CCL3	Chemokine (C-C motif) ligand 3
1.0	2.55	0.64	0.70	CXCL11	Chemokine (C-X-C motif) ligand 11
1.0	2.30	2.51	1.35	CCL2	Chemokine (C-C motif) ligand 2
1.0	1.00	0.50	0.49	IL18R1	Interleukin 18 receptor 1
1.0	0.98	2.12	2.05	IL1B	Interleukin 1, beta
1.0	0.92	2.36	1.83	IL1B	Interleukin 1, beta

### Quantification of ginsenosides by HPLC analysis

**Figure 3:**
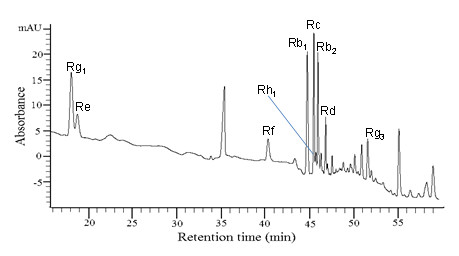
**High performance liquid chromatography analysis of PGSE**. The separation was done by using a reversed-phase column Lichrospher 100 C_18 _reversed-phase and the detection wavelength was set at 203 nm for all ginsenosides. The gradient program consisted of two solvents (A) water and (B) acetonitrile at a flow of 1 mL/min as follows: 0–6 min, 21–22% B; 6–7 min, 22–23% B; 7–25 min, 23–24% B; 25–30 min, 24–30% B; 30–40 min, 30–32% B; 40–45 min, 32–50% B; 45–60 min, 50–65% B; 60–61 min, 65–100% B; and 61–65 min, back to 21% B before the next injection for analysis. Twenty micrograms of PGSE was injected each time.

Since ginsenosides are major active ingredients in ginseng, we examined the composition of ginsenosides in PGSE by HPLC analysis and the results are shown in Figure 3. The calibration curves of the standard solutions containing 0.5–6.5 μg of each ginsenosides were plotted as the peak area versus the amount of selected ginsenosides. Individual ginsenosides from the PGSE were identified and quantified by retention time and peak areas, respectively, as compared to the commercially available pure standards. Nine ginsenosides including Rb_1_, Rb_2_, Rc, Rd, Re, Rf, Rg_1_, Rg_3 _and Rh_1 _were identified in the PGSE. The amount, concentration and the percentage of each ginsenoside in 3 mg of PGSE are shown in Table 1.

### Differential effects of ginsenosides on TNF-α stimulated-U937 cells

**Figure 4:**
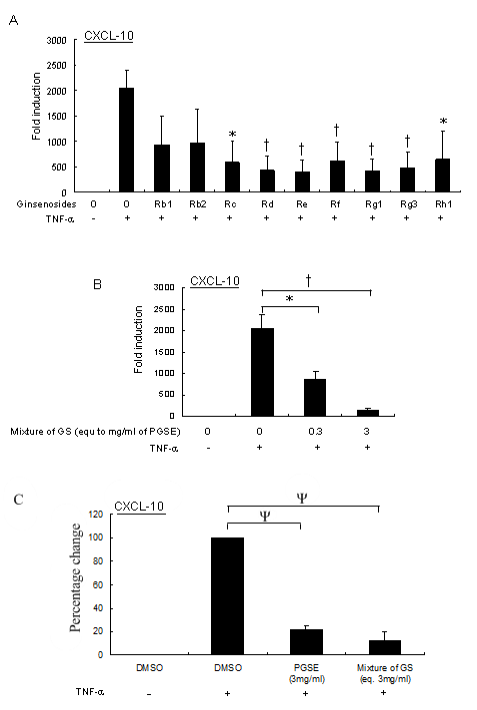
**Suppressive effects of ginsenosides on U937 cells stimulated with TNF-α**. (A) Nine ginsenosides were standardized to concentrations in the PGSE at 3 mg/ml according to Table 1. U937 cells were treated with ginsenosides for 24 hours following with 20 units/ml TNF-α stimulation for 2 hours, and the transcription of CXCL-10 was measured by quantitative RT-PCR as described in Methods. (B) Ginsenosides including Rb_1_, Rb_2_, Rc, Rd, Re, Rf, Rg_1_, Rg_3 _and Rh_1 _were pooled together to investigate the combinatorial effect of the nine ginsenosides on CXCL-10 transcription following TNF-α stimulation by using quantitative RT-PCR. (C) Comparable inhibitory effects of the ginseng extract (PGSE) and the mixture of individual ginsenosides on CXCL-10 transcription. U937 cells were treated with 3 mg/ml of PGSE or the mixture of GS (that is equivalent to 3 mg/ml of PGSE) for 24 hours following with 20 units/ml of TNF-α stimulation for another 2 hours. The transcription of CXCL-10 was measured by quantitative RT-PCR as described in Methods. Values represent the average ± SD of three independent experiments and statistically analyzed by two tailed, paired *t*-test. ψ: p < 0.005; †: p < 0.01; *: p < 0.05. GS, ginsenosides; PGSE, 70% ethanol-water extracts of ginseng.

To investigate whether the CXCL-10 suppressive effect by 3 mg of PGSE was due to a specific ginsenoside, U937 cells were treated with individual ginsenosides using the amount as listed in Table 1 for 24 hours and followed by TNF-α stimulation. The level of CXCL-10 transcription was measured by quantitative RT-PCR. With the exception of ginsenosides Rb_1 _and Rb_2_, our results showed that the CXCL-10 transcription were significantly inhibited by ginsenosides including Rd, Re, Rf, Rg_1 _and Rg_3 _(p < 0.01), as well as by Rc and Rh_1 _(p < 0.05; Figure 4A). However, it is noted that the extent of the suppressive effect of individual ginsenosides on CXCL-10 transcription was still less than that of the PGSE mixture. As ginsenosides accounted for only 18.8% of PGSE by weight; and thus other constituents present in significant concentrations may modulate the activity of the ginsenosides.

We then investigated the combinatorial effect of the nine ginsenosides on TNF-α induced-CXCL-10 transcription. The nine ginsenosides were standardized to concentrations in the PGSE at 3 mg/ml according to Table 1. Moreover, we included a 10-fold dilution ginsenoside mixture to examine the dose-dependent effect on CXCL-10 suppression. Interestingly, the suppressive effect of the reconstituted mixture of ginsenosides at a dose equivalent to 3 mg/ml of PGSE on TNF-α induced-CXCL-10 transcription was comparable to the PGSE treatment (Figures 1A and 4B). Moreover, the suppressive effect of the mixture of ginsenosides occurred in a dose-dependent manner (Figure 4B). To examine the comparable inhibitory effects of PGSE and the mixture of ginsenosides, we measured the percentage change of TNF-α induced-CXCL-10 mRNA after the pretreatment of 3 mg/ml of PGSE, or the mixture of ginsenosides that were equivalent to their corresponding amounts in 3 mg/ml of PGSE. Our results showed that the mixture of ginsenosides gives comparable inhibition of CXCL-10 transcription to those with PGSE (p < 0.005, Figure 4C), but the percentage change of CXCL-10 mRNA between these two treatments was not statistically significance (p > 0.1). Hence, our results indicated that the suppressive effect of PGSE on TNF-α induced-CXCL-10 transcription can be due to the combinatorial effect of ginsenosides.

### Inhibition of TNF-α-activated signal transduction pathways by PGSE

**Figure 5:**
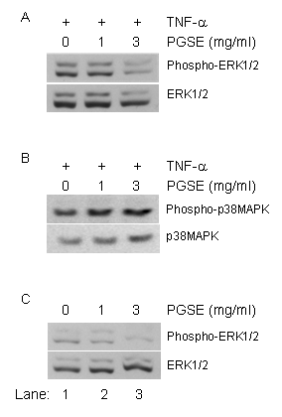
**Inhibition of MAP kinases activation after PGSE treatment**. U937 cells were treated with PGSE (1 or 3 mg/ml) for 24 hours followed by 20 units/ml TNF-α stimulation for 2 hours. Whole cell protein lysate was analyzed by Western analysis using (A) anti-phospho ERK1/2 antibodies; and (B) anti-phospho p38MAPK antibodies as described in Methods. (C) Cell lysate with PGSE treatment only was analyzed by anti-phospho ERK1/2 antibodies. Equal amount of protein loading in the blot was shown by staining the immunoblot with anti-ERK1/2 or anti-p38MAPK antibodies. PGSE, 70% ethanol-water extracts of ginseng.

To investigate the underlying mechanisms of the suppressive effect of the PGSE on CXCL-10 induction, we measured the activities of MAP kinases, including ERK1/2 and p38MAPK, by Western analysis. Intense activation of phospho-ERK1/2 and phospho-p38MAPK was detected after TNF-α stimulation (lane 1, upper panel, Figure 5A and 5B). However, the level of ERK1/2 phosphorylation was decreased with PGSE pretreatment (lanes 2–3, upper panel, Figure 5A). In contrast, the PGSE did not show inhibitory effects on TNF-α activated phospho-p38MAPK activity (lanes 1–3, upper panel, Figure 5B). Interestingly, we found that PGSE inhibited the basal level of ERK1/2 phosphorylation at 1 or 3 mg/ml (lanes 2 and 3, Figure 5C). Equal loading amount of the proteins in the blot was shown by staining the immunoblot with anti-ERK1/2 antibodies (low panel, Figure 5C). In addition to the MAPK signalling pathways, we examined the effects of PGSE on the nuclear translocation of transcription factor NF-κB in the TNF-α treated cells by Western analysis. However, the PGSE did not inhibit the nuclear translocation of p50 and p65 subunits of NF-κB in the TNF-α treated-cells suggesting that the PGSE targets the ERK1/2 signalling pathways (data not shown).

## Discussion

Ginseng is one of the most commonly used herbal medicines in China, Asia and Western countries. Studies have shown a wide range of beneficial effects of ginseng against human diseases [25]. The potential therapeutic effects of ginseng have been attributed to its immunostimulatory, anti-oxidant and anti-inflammatory activities. In this study, we used human promonocytic U937 cells to investigate the modulatory effects of ginseng in cellular response to TNF-α-mediated inflammation. By using the genechip approach, we obtained a global gene expression profile in monocytic cell model following different experimental treatments. Our genechip results showed a potent suppressive effect of the PGSE on the expression of TNF-α-inducible genes including CXCL-10. These results have been validated by using quantitative RT-PCR and ELISA. Moreover, nine ginsenosides were identified in our ginseng extract by using HPLC analysis. Interestingly, other groups have reported the anti-inflammatory activity of these ginsenosides. Our results showed that seven out of nine ginsenosides could significantly inhibit TNF-α-induced CXCL-10 expression in U937 cells. However, the suppressive effect of individual ginsenosides on CXCL-10 induction was less than that of the mixture of ginsenosides or PGSE alone. Furthermore, we found that the CXCL-10 suppressive effect correlates with the inactivation of the ERK1/2 signalling pathways by PGSE.

The immunomodulatory effects of ginseng or ginsenosides have been reported in *in vivo *and *in vitro *studies. Kim *et al*. showed that *Panax ginseng *enhances the recovery of natural killer (NK) cell functions in cyclophosphamide-treated mice, and provides protection against infection with *Listeria monocytogenes *[26]. Ginseng radix extracts induce production of TNF-α and IFN-γ in murine spleen cells and peritoneal macrophages via toll-like receptor (TLR)-4 [5]. Additionally, Ginsenan S-IIA, a component of acidic polysaccharide of *Panax ginseng*, is a potent inducer of IL-8 in human monocytes and THP-1 cells [7]. In contrast, ginseng or ginseng extract have been shown to have anti-inflammatory effects such as suppressing the expression of proinflammatory cytokines or mediators. For instance, ginsan, a polysaccharide extracted from *Panax ginseng*, protects mice from lethality induced by *Staphylococcus aureus *and such effect was associated with suppression of proinflammatory cytokines production including TNF-α, IL-1β, IL-6, IL-12, IL-18 and IFN-γ [9]. Moreover, 20(S)-Protopanaxatriol, one of the major metabolites of ginsenosides, inhibits the increase in iNOS and COX-2 expressions following LPS stimulation through inactivation of NF-κB [13]. The diverse immunologic effects of ginseng may be due to multiple effects of the ginsenosides or its other active components. Therefore, comprehensive studies of ginseng and its constituents are still needed to provide detailed understanding of their actions in humans.

Since our study is focused on immunomodulation, only the list of cytokines or cytokine-regulated genes is reported in Table 2. Here, the PGSE can cause a potent inhibition on the transcription of TNF-α inducible genes including CXCL-10, G protein-coupled receptor 84, TNF-α induced-protein 6, IκB-alpha, IκB-zeta and phosphodiesterase 4B (Table 2). Interestingly, those genes inhibited by PGSE have been shown to be expressed in TNF-α mediated-inflammatory diseases [15,27-29]. Therefore, it is plausible that ginseng down regulates TNF-α mediated inflammation through suppressing the production of inflammatory mediators in monocytes or macrophages. However, it seems that this PGSE preparation did not contain potent cytokine inducing factors. As previous reports showed that the immunostimulating components such as polysaccharides of ginseng extracts come from the ethanol insoluble fraction [7,30,31], this component appears to have been excluded or its biological activity was attenuated by constituents in the extract we studied.

CXCL-10 is an important chemokine downstream of TNF-α signalling pathways and a well-documented mediator of inflammation. CXCL-10 initiates its biological functions through binding to its high affinity receptor CXCR-3 leading to recruitment of the activated effector lymphocytes including CD4+ and CD8+ T cells as well as NK cells to the site of infection or injury [32]. Similar to TNF-α, the uncontrolled production of CXCL-10 also is associated with the pathogenesis of acute and chronic inflammatory diseases including intrahepatic inflammation during chronic HCV infection, atherosclerosis, inflammatory bowel disease, and multiple sclerosis as well as tumorigenesis and metastasis [33-37]. In our study, the PGSE or chemically defined mixture of its constituent ginsenosides showed potent inhibitory effects on TNF-α-stimulated CXCL-10 expression (Figure 4C) suggesting a specific anti-inflammatory property of ginseng.

Ginsenosides belong to a family of steroidal saponins that are believed to be responsible for the pharmacological effects of ginseng. About 30 different ginsenosides have been isolated and identified from *Panax ginseng*. The two major groups of ginsenosides are panaxadiol and panaxatriol. The panaxadiol group contains Rb_1_, Rb_2_, Rc, Rd and Rh_2 _whereas the panaxatriol group contains Re, Rf, Rg_1_, Rg_2,_Rg_3 _and Rh_1_. Previous studies have shown different properties of ginsenosides among each other, and differential effects of ginsenosides panaxadiol and panaxatriols have been found in inflammatory diseases [38]. Here, we found that both of the panaxadiol and panaxatriol groups of ginsenosides showed similar inhibitory effects on TNF-α-induced CXCL-10 production. Additionally, the inhibitory effects could be due to complementary or collective effect of ginsenosides mixtures instead of a single ginsenoside. Another possible explanation is stereoisomerism of natural and synthetic compounds since the source of ginsenosides is different from the ginseng extract. Similar phenomenon has been reported by another group recently [39].

Following the activation of TNF-α signalling pathways, the downstream MAPK cascades and transcription factors, NF-κB and AP-1, are activated to induce gene transcription. Previous studies have shown that NF-κB and/or MAPK signalling cascades play critical roles in acute and chronic inflammatory diseases. Here our result showed that the PGSE inhibited the basal level of ERK1/2 phosphorylation at 1 or 3 mg/ml (Figure 5C). This observation is in agreement with the effect of PD98059, a known inhibitor of ERK1/2, on the suppression of TNF-α-induced CXCL-10 transcription (not shown). In contrast, the PGSE did not show any effect on TNF-α-induced activation of p38MAPK and NF-κB. These results suggest that PGSE inhibited CXCL-10 expression by perturbing MAPK signalling cascades.

## Conclusion

In conclusion, the results of this study provide evidence that ginseng can suppress TNF-α-inducible cytokines and signalling proteins in promonocytic cells. The suppressive effect of the reconstituted mixture of individual ginsenosides on TNF-α induced-CXCL-10 transcription was comparable to that of the PGSE treatment. Moreover, ginseng down regulated CXCL-10 expression by suppressing TNF-α-induced ERK1/2 activation. Thus, ginseng may exert its anti-inflammatory properties by targeting at different levels of the TNF-α signalling pathways. Further studies will be needed to examine the potential beneficial effects of ginsenosides in the management of acute and chronic inflammatory diseases in humans.

## Competing interests

ASYL has received grants for basic science research from Purapharm International since 2007.

## Authors' contributions

DL participated in study design, data acquisition, interpretation and manuscript writing. CY participated in study design, chemical analysis and data interpretation. SC participated in biomolecular assays and data interpretation. JL, JR and GC participated in study design and interpretation of results. AL designed the study and led the data interpretation and manuscript writing. All authors have read and approved the final manuscript.
